# Benchmark of Available Explicit Solvent Models in CHARMM36m to Characterize Glycosaminoglycans

**DOI:** 10.1021/acs.jpcb.5c04919

**Published:** 2025-09-24

**Authors:** P. A. Wesołowski, D. J. Wales, K. K. Bojarski

**Affiliations:** † Yusuf Hamied Department of Chemistry, 2152 University of Cambridge, Lensfield Road, Cambridge CB2 1EW, U.K.; ‡ Department of Physical Chemistry, Gdansk University of Technology, Narutowicza 11/12, Gdansk 80-233, Poland; § Center for Functional Protein Assemblies, Technical University of Munich, Ernst-Otto-Fischer-Straße 8, Garching 85747, Germany

## Abstract

Heparin is a highly sulfated glycosaminoglycan (GAG) with essential roles in anticoagulation, angiogenesis, cell signaling, and host–pathogen interactions, mediated largely through electrostatic binding to diverse protein targets. While the TIP3P water model is commonly used in molecular simulations of GAGs, the influence of solvent representation on HP structure within the CHARMM36m force field remains poorly understood. Here, we report 5 μs molecular dynamics simulations of an HP dodecamer in five explicit solvent models: TIP3P, TIP4P, TIP5P, SPC/E, and OPC. TIP3P and SPC/E yield stable HP conformations, whereas TIP4P, TIP5P, and OPC introduce greater structural variability. Comparison with GLYCAM06 reveals that CHARMM36m preserves global HP architecture but differs in sampling specific glycosidic linkages. These findings highlight the critical impact of water model choice on GAG conformational dynamics and offer practical guidance for the accurate simulation of sulfated carbohydrates.

## Graphical abstract

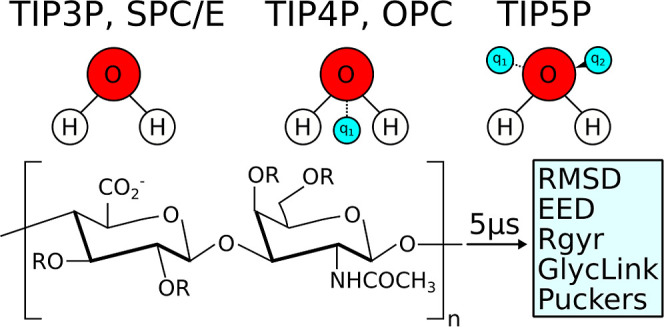

## Introduction

Heparin (HP), a sulfated polysaccharide, is primarily composed of repeating GlcNS­(6S)–IdoA­(2S) disaccharide units.^1^ Its carbohydrate chain typically contains 10–50 disaccharide units, yielding an average molecular weight of approximately 75 kDa.^2^ Alongside chondroitin sulfate, dermatan sulfate, keratan sulfate, heparan sulfate, and hyaluronic acid, HP belongs to the GAGs, a family of linear, anionic carbohydrates found in the extracellular matrix and acidic lysosomes.^3,4^ HP plays critical biological roles in angiogenesis, anticoagulation, cell growth and development, and antiviral defense.^5^ It also contributes to protease storage and modulates wingless signaling when associated with a protein core.^6^ These functions are largely mediated by electrostatic interactions with diverse protein partners, including antithrombin,^7^ fibroblast growth factors (FGFs),^8^ vascular endothelial growth factor (VEGF),^9^ and chemokines.^10^ Consequently, HP has been implicated in therapeutic strategies for thrombosis,^11^ cancer metastasis,^12^ sepsis,^13^ and inflammation-related diseases.^14^

Despite advances in methodology, in silico investigations of GAG-containing systems remain challenging. Major difficulties arise from the high conformational flexibility of glycosidic linkages^15^ and ring puckering,^16^ as well as the capacity of GAGs to adapt multiple binding poses at a single protein site with comparable free energies.^17–19^ Moreover, the limited number of experimentally resolved GAG structures complicates validation and hinders the application of machine learning approaches.^20,21^ Interactions with proteins are strongly electrostatic in nature, and optimizing the ionic environment alongside the solvent is therefore crucial.^22,23^ This aspect will be explored in future work. Another significant challenge is the treatment of solvent,^24^ which plays a critical role in modulating protein–GAG interactions. In computational studies, solvent can be modeled either implicitly or explicitly.

Implicit solvent models are based on the Poisson–Boltzmann equation^25^ and its generalized born approximation,^26^ which treat the solvent as a continuous dielectric medium. This approach reduces computational cost by averaging solvent effects spatially and neglecting solvent–solvent interactions. Consequently, implicit models are widely used^27,28^ in the calculation of electrostatic potential isosurfaces via the Poisson–Boltzmann surface area (PBSA) method,^29^ in molecular docking protocols,^30–34^ and in molecular mechanics Poisson–Boltzmann surface area (MM-PBSA) estimations of binding affinities.^35,36^

Alternatively, in explicit solvent models, individual water molecules are explicitly represented, typically using three-site (e.g., TIP3P; transferable intermolecular potential with 3 points), four-site (e.g., TIP4P; transferable intermolecular potential with 4 points), or five-site (e.g., TIP5P; transferable intermolecular potential with 5 points) descriptions that differ in the treatment of charge distribution and molecular geometry. The TIP3P model is by far the most frequently employed in protein–GAG simulations,^37–40^ offering a balance between computational efficiency and reliability due to its simple three-point design.

Comparative studies of solvent models in GAG simulations are limited. Marcisz et al.^41^ assessed unbound HP decasaccharide (dp10) in both implicit and explicit solvents, concluding that implicit models poorly reproduced experimental ring puckering conformations, whereas explicit solvents offered improved accuracy. In particular, TIP3P promoted a U-shaped curvature in HP, in which reducing and nonreducing ends interacted via sodium counterions, suggesting that longer simulations with more advanced models may yield more realistic electrostatic environments. Among the solvents tested, OPC (optimal point charge) and TIP5P best reproduced both local and global features of HP. More recently, Anila and Samsonov^42^ studied several GAG–protein complexes—including HP–FGF2, chondroitin 4-sulfate–cathepsin K, and hyaluronic acid–CD44—and found that structural and energetic features were better captured using explicit solvents. Interestingly, the effect of solvent choice diminished with increasing protein–GAG binding affinity. Notably, both of these studies employed the GLYCAM06j force field, and to date, no comparable assessment has been reported for CHARMM36m.

In the present study, we investigate the influence of five explicit solvent models—TIP3P, TIP4P, TIP5P, SPC/E (extended simple point charge), and OPC (Figure 1)—on the conformational behavior of a heparin dodecamer (dp12) simulated using the CHARMM36m force field. We performed 5 μs molecular dynamics simulations for each solvent model and analyzed the resulting trajectories using several structural descriptors, including root-mean-square deviation (RMSD), radius of gyration, end-to-end distance, ring puckering, and glycosidic linkage conformations. By comparing the outcomes across different solvent models, we identify systematic variations that provide practical insights into solvent model selection for the accurate modeling of sulfated GAGs in biomolecular simulations.

## Methods

### Analyzed Solvent Models

Explicit solvent models treat water molecules in full atomistic detail, differing primarily in the number and spatial arrangement of interaction sites. Common models include TIP3P,^43^ TIP4P,^44,45^ and TIP5P,^46^ which vary in geometry and charge distribution to reproduce key physical properties of water. These models aim to strike a balance between computational cost and accuracy in capturing bulk water behavior. TIP3P is widely adopted in biomolecular simulations due to its simplicity and efficiency.

In TIP3P, partial charges reside on the hydrogen atoms, while van der Waals interactions are applied between oxygen atoms. The TIP4P model introduces a massless charge site displaced from the oxygen atom to improve dielectric properties, while TIP5P adds two lone-pair interaction sites to better represent the tetrahedral structure of water and its anomalous density near 4 °C. The dipole moments of TIP3P, TIP4P, and TIP5P are 2.35, 2.18, and 2.29 D, respectively. In TIP5P, the oxygen atom carries no charge, and nonbonded interactions are governed solely by a Lennard–Jones potential between oxygen atoms, with a value of σ_0_ = 3.12 Å and ε_0_ = 0.16 kcal mol^–1^. The interaction energy between two water molecules, *a* and *b*, is given by

1 $$E_{ab}=\sum_{ij}\frac{q_{i}q_{j}e^{2}}{r_{ij}}+4\varepsilon_{0}\left[{\left(\frac{\sigma_{0}}{r_{OO}}\right)}^{12}-{\left(\frac{\sigma_{0}}{r_{OO}}\right)}^{6}\right]$$

where *i* and *j* index the interaction sites on molecules *a* and *b*, and *r*_OO_ is the oxygen–oxygen separation. The Lennard–Jones potential models short-range repulsion and long-range dispersion between nonbonded atoms, with ε and σ characterizing the potential depth and effective molecular diameter, respectively. This potential is essential in reproducing the structural and dynamic features of liquid water.

Improved variants such as TIP4P/2005 ^45^ and TIP4P/Ew^47^ offer enhanced agreement with experimental observables. The SPC/E model^48^ modifies the TIP3P charge distribution and includes a polarization correction to improve performance across dynamic and thermodynamic properties. The OPC model^49^ refines site placement and interaction parameters to achieve high fidelity with experimental data over a broad range of conditions.

### Molecular Dynamics

In the following study, we simulated HP dodecamer (PDB ID: 1HPN). Input files for MD simulations in CHARMM36m force field were prepared with application of CHARMM-GUI.^50^ In the first step, HP was solvated in an octahedral periodic box of each solvent with a layer of water molecules of 6 Å from the border of the periodic box to the solute and neutralized with counterions (Na^+^) described in an all-atom representation using the standard parameters provided by CHARMM-GUI.^50^ For each system, energy minimization was carried out in one step. Positional restraints were imposed on backbone atoms, side chains and dihedral angles with force constants of 400, 40, and 4 kJ mol^–1^ rad^2^. A steepest descent algorithm for energy minimization with the tolerance of 10^4^ kJ mol^–1^ nm was employed. In the energy minimization step, hydrogen bonds were converted to rigid holonomic constraints with the LINCS algorithm.^51^ Next, the equilibration step was carried out with the same positional restraints as in energy minimization. Equilibration simulation was performed in the *NVT* ensemble for 125 ps at 300 K and temperature coupling using a Nose–Hoover extended ensemble. Finally, five 5 μs productive MD runs (one for each solvent model) for each complex were carried out in an *NPT* ensemble. Nose–Hoover and Parrinello–Rahman algorithms were used for temperature and pressure control, respectively. The structures were written every 100 ps, which produced 5 × 10^3^ in total per simulation used for further analysis.

### Structural Analysis

Trajectory analyses were performed to characterize the conformational dynamics of HP. The RMSD was calculated relative to the first simulation frame using the gmx rms module from the GROMACS package.^52^ The RMSD matrix was generated via Biopython^53^ following structural alignment. End-to-end distances (EEDs) were computed using the DISTANCE module of PLUMED,^54^ defined as the distance between the centers of mass (COM) of the terminal monosaccharide units. The radius of gyration (*R*_g_) was determined using gmx gyrate module from the GROMACS package.^52^

Glycosidic torsions were analyzed in accordance with the definitions of Sattelle et al.,^16^ specifically the dihedral angles O_5(*n*+1)_–C_1*n*+1_–O_4*n*_–C_4*n*_ and C_1*n*+1_–O_4*n*_–C_4*n*_–C_3*n*_, where *n* denotes the residue index. These were computed using the TORSION module of PLUMED.^54^ Monosaccharide ring conformations were classified based on the dihedral angles γ (C_1_–C_2_–C_3_–C_4_) and δ (C_1_–O_5_–C_5_–C_4_), following our previous work (Figure 2).^15^ A range of ±23° around reference γ and δ values was used to assign four principal puckering states: ^1^C_4_, ^4^C_1_, ^2^S_O_, and ^1^S_3_.

### Comparison with ff14SB/GLYCAM06j

Structural properties obtained in this study were compared with the results reported for HP dp10 using the ff14SB/GLYCAM06j force field by Marcisz et al.^41^ In addition to *R*_g_, EED, glycosidic linkage, and ring puckering distributions, RMSD analyses were carried out using the cpptraj module of the AMBER package,^55^ referenced to the initial MD frame. RMSD matrices were computed following the same protocol as described in the Structural Analysis section (see Figures 1 and 2).

**Figure 1:**
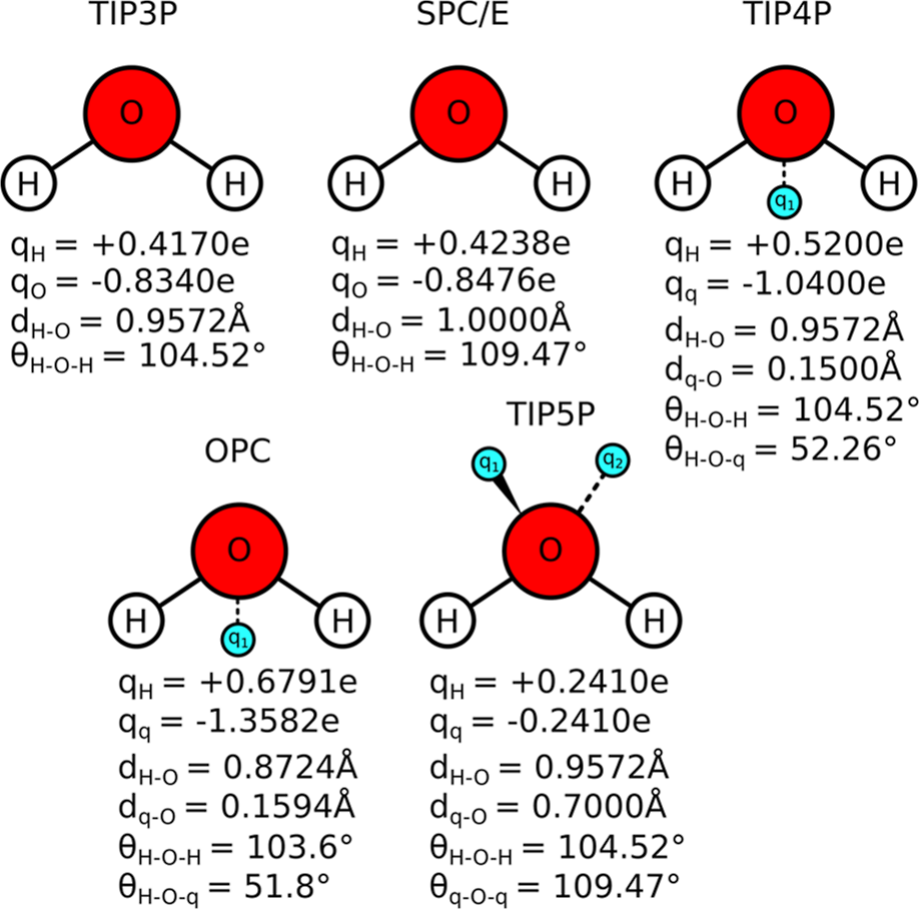
Schematic representation of the solvent models employed in this study and their MD simulation parameters.

**Figure 2:**
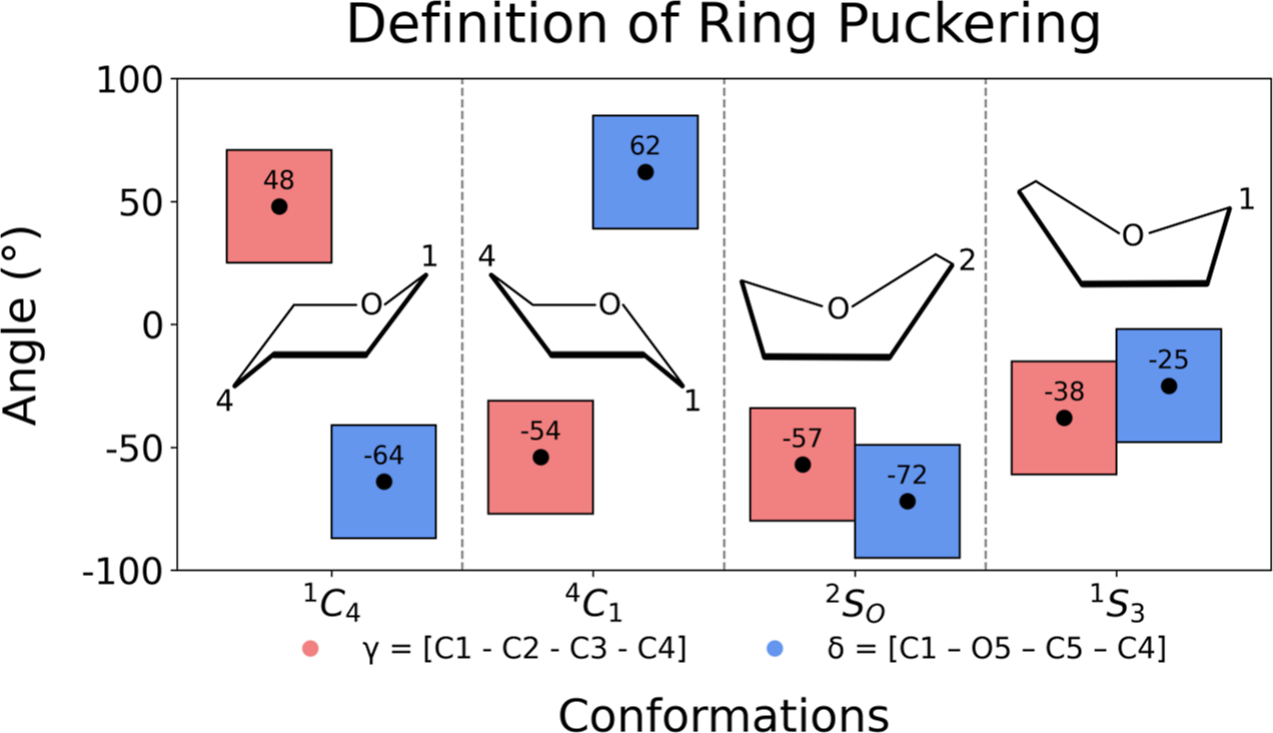
The definitions of monosaccharide ring conformations based on the values of γ and δ dihedrals and schematic representation of pyranose ring in these conformations.

## Results and Discussion

### Root-Mean-Square Deviation Evaluation

The RMSD values of HP dp12 were evaluated relative to the initial structure over 5 μs MD simulations in five explicit solvent models. Overall, the mean and standard deviation values were comparable across all models (*m* = 5.38–5.76; σ = 0.44–0.74). However, closer inspection reveals a trend where increased solvent model complexity correlates with greater deviation from the initial structure (Figure 3).

**Figure 3:**
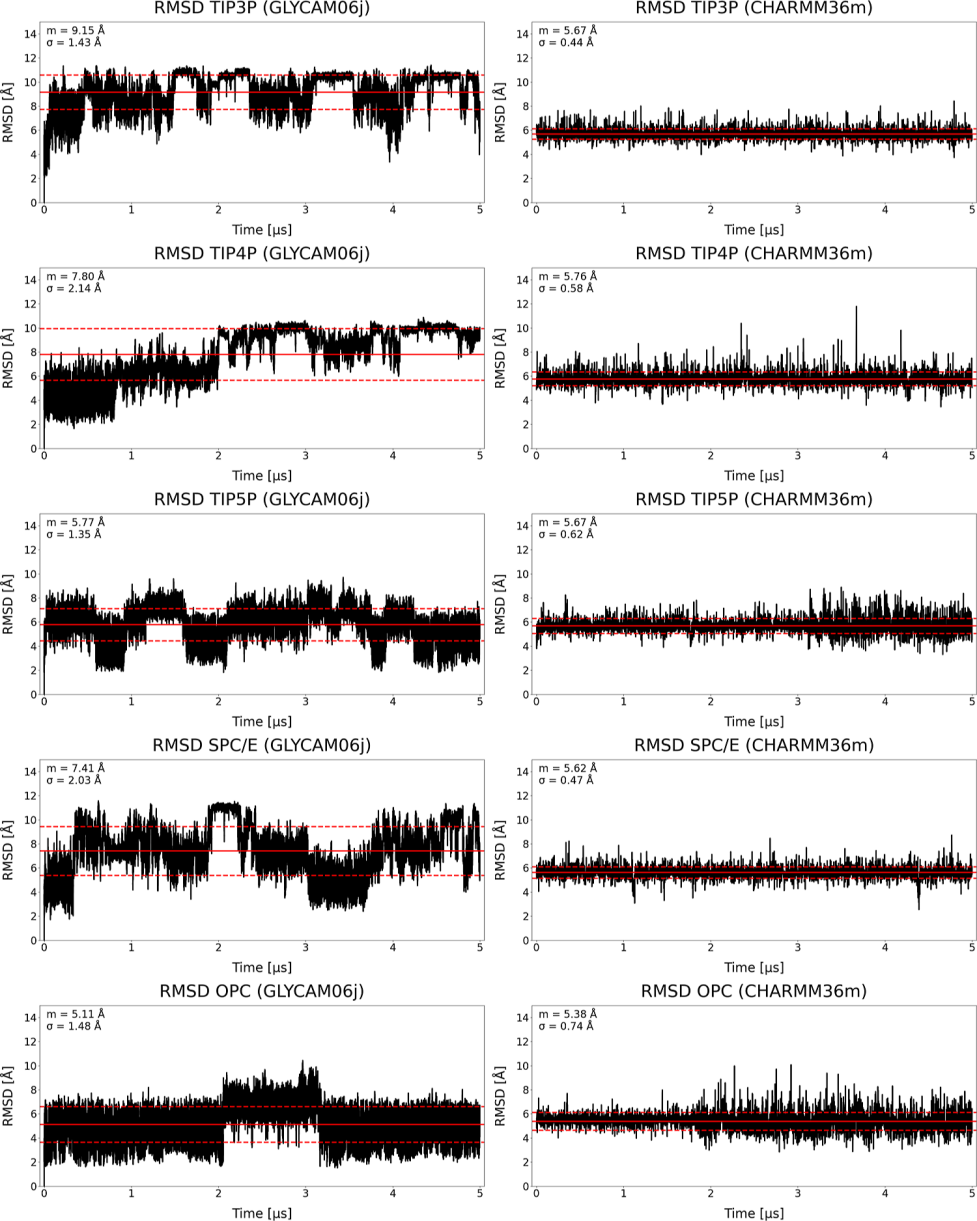
RMSD results of HP dp10 in GLYCAM06j and HP dp12 in CHARMM36m, solvated in selected explicit solvent models.

Among the tested solvents, TIP3P and SPC/E showed lower RMSD fluctuations and smaller standard deviations, indicating more consistent structural environments for HP. This apparent stability likely reflects the simpler parametrization of these models, which may restrict HP conformational dynamics. In contrast, TIP4P exhibited numerous sharp RMSD peaks, suggesting spontaneous structural transitions potentially driven by its enhanced dipole description. For OPC and TIP5P, increased RMSD variability was observed around 2 and 3 μs, respectively, implying greater conformational sampling. The enhanced polarity and complexity of these models may afford a more dynamic solvation environment that promotes HP flexibility.

Notably, the RMSD values reported here are generally lower than those from a previous study on HP dp10 with the GLYCAM06j force field (*m* = 5.11–9.15; σ = 1.35–2.14).^41^ Based on RMSD, OPC and TIP5P showed the highest structural stability, reflected in the lowest *m* and σ values, while the three-point models allowed for greater flexibility. This suggests that CHARMM36m, although permitting conformational changes, does so within a more restrained regime than GLYCAM06j. These differences likely arise from how the force fields treat HP flexibility and solute–solvent interactions, with CHARMM36m offering a more balanced and conservative conformational landscape.

To further examine HP structural variability, RMSD matrices were constructed by aligning all MD-generated structures and computing pairwise RMSD values (Figure 4). For TIP3P, the matrix revealed uniformly low RMSD values, indicating that HP dp12 maintains a highly stable conformation throughout the 5 μs simulation. This observation aligns with the low mean and standard deviation reported in the time-resolved RMSD analysis. Similarly, the SPC/E matrix showed predominantly low values, though with slightly greater variability over time, consistent with the modestly higher RMSD statistics and suggesting minor conformational drift.

**Figure 4:**
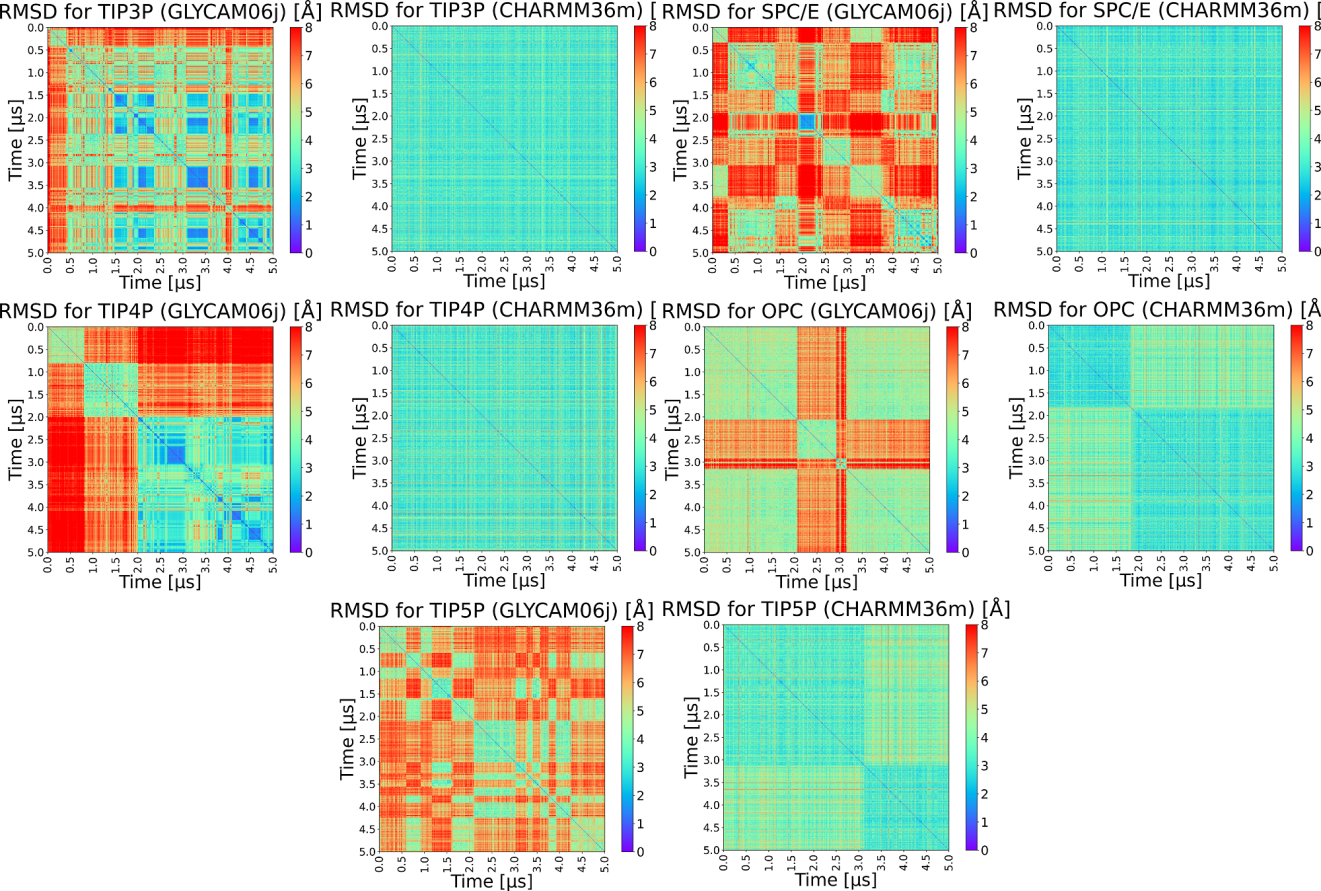
RMSD matrices of HP dp10 in GLYCAM06j and HP dp12 in CHARMM36m structures from MD simulations in selected explicit solvent models.

In contrast, TIP4P exhibited notable structural transitions, evident from multiple orange and red regions in the matrix. These correspond to transient conformers with elevated RMSD, confirming the increased conformational flexibility inferred from the RMSD time series. For the OPC solvent, two distinct conformational clusters were observed: the first dominating up to 1.8 μs, and the second persisting thereafter. Interspersed high-RMSD regions further suggest the occurrence of transient conformational rearrangements. TIP5P showed a similar bifurcation, with a major conformational transition at approximately 3.1 μs, accompanied by increased structural heterogeneity—more pronounced than in any other solvent model studied with CHARMM36m.

Parallel RMSD matrix analyses were performed for trajectories obtained with the GLYCAM06j force field. Here, structural differences were more pronounced overall. Among the tested solvents, OPC again provided the most stable behavior, with only two major conformational clusters throughout the trajectory. TIP4P also yielded relatively constrained dynamics, with three identifiable clusters, the last of which—emerging in the second half of the simulation—displayed high internal similarity and low RMSD. TIP3P followed a comparable pattern, albeit with a higher number of conformational states. The greatest structural diversity was observed in SPC/E and TIP5P, consistent with enhanced conformational heterogeneity.

Taken together, these results support the conclusion that HP conformational stability is highest in OPC for both CHARMM36m and GLYCAM06j simulations. In particular, OPC appears well-suited for preserving native-like GAG features, especially when used with the GLYCAM06j force field.

### End-to-End Distance and Radius of Gyration Assessment

Molecular shape and compactness were further assessed via EED and *R*_g_, two key descriptors of GAG conformational behavior. These metrics are particularly informative given that specific glycan shapes, such as the helical conformation observed in FGF–HP complexes, can be functionally significant.^56^ Across all solvent models examined, mean EED values ranged from 34.39 to 35.65 Å, with standard deviations between 4.73 and 4.97 Å (Figure 5). This narrow distribution indicates that HP maintains a broadly similar level of extension regardless of solvent, with moderate fluctuations in flexibility.

**Figure 5:**
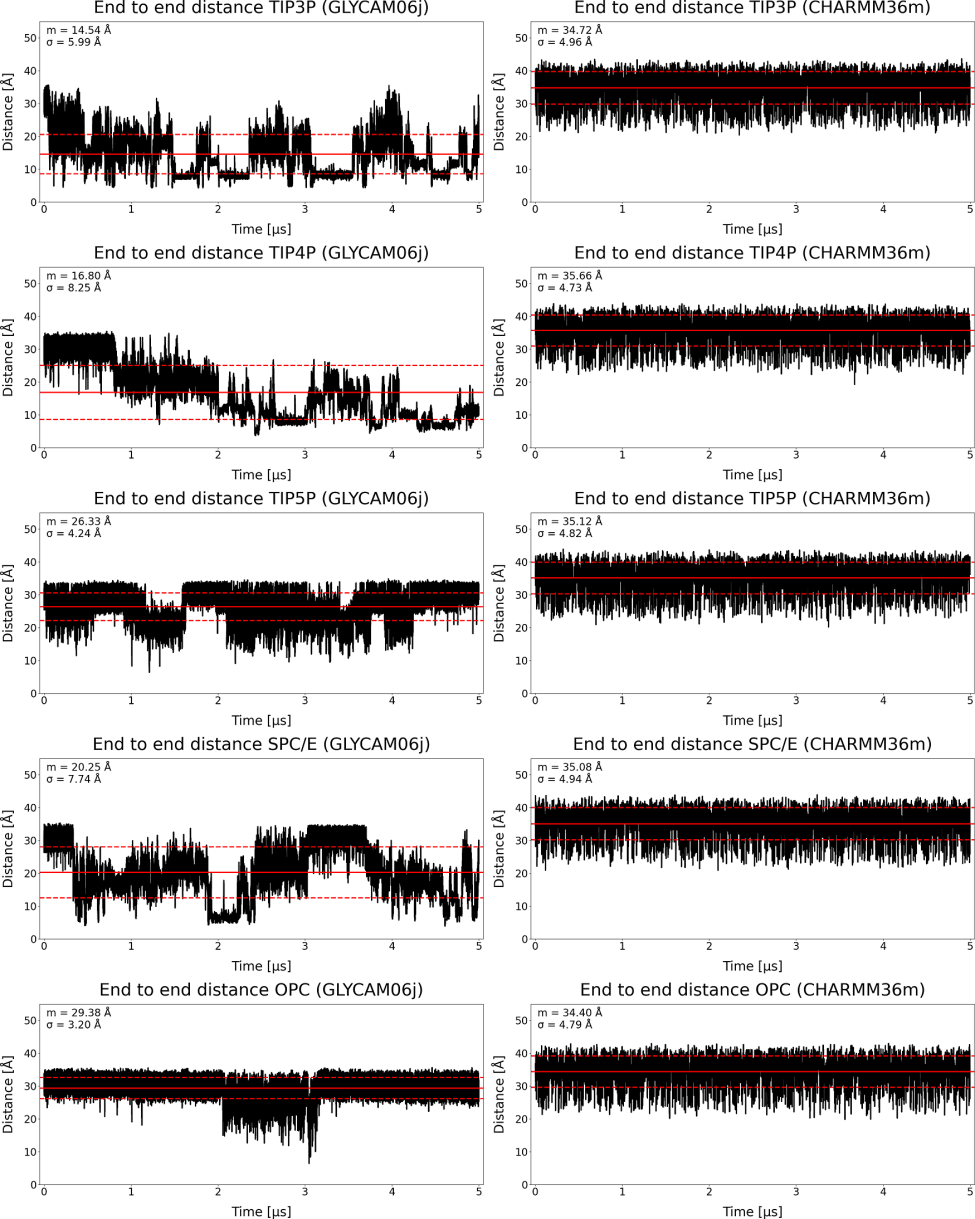
EED results of HP dp10 in AMBER and HP dp12 in CHARMM, solvated in selected explicit solvent models.

In contrast to the results of Marcisz et al.,^41^ where HP dp10 displayed marked bending in TIP3P, SPC/E, and TIP4P solvents, manifested as reducing and nonreducing ends coming into proximity and *R*_g_ values falling below 10 Å, no such conformations were observed here. Instead, the present data indicate greater conformational stability, particularly in TIP5P and OPC, in agreement with RMSD trends. Notably, EED values obtained with CHARMM36m in explicit solvent are consistently lower than those reported for GLYCAM06j in implicit solvent, suggesting that the HP chain remains moderately compact and retains intrachain contacts regardless of the explicit solvent model used.

Similar to EED, the calculated *R*_g_ values remained consistent across all solvent models examined (Figure 6). Mean values ranged from 14.89 to 15.26 Å, with standard deviations between 0.42 and 0.56 Å. TIP3P and SPC/E models yielded compact and stable conformations throughout the simulations, with minimal fluctuations. The two four-site models showed comparable behavior, although structures with slightly lower *R*_g_ values occurred more frequently. In TIP4P, such structures persisted across the full trajectory, whereas in OPC they appeared to coincide with the emergence of a secondary conformational cluster, as also observed in the RMSD matrix analysis. A similar trend was seen in TIP5P, where distinct minima in *R*_g_ correlated with structural transitions into an alternative conformational cluster.

**Figure 6:**
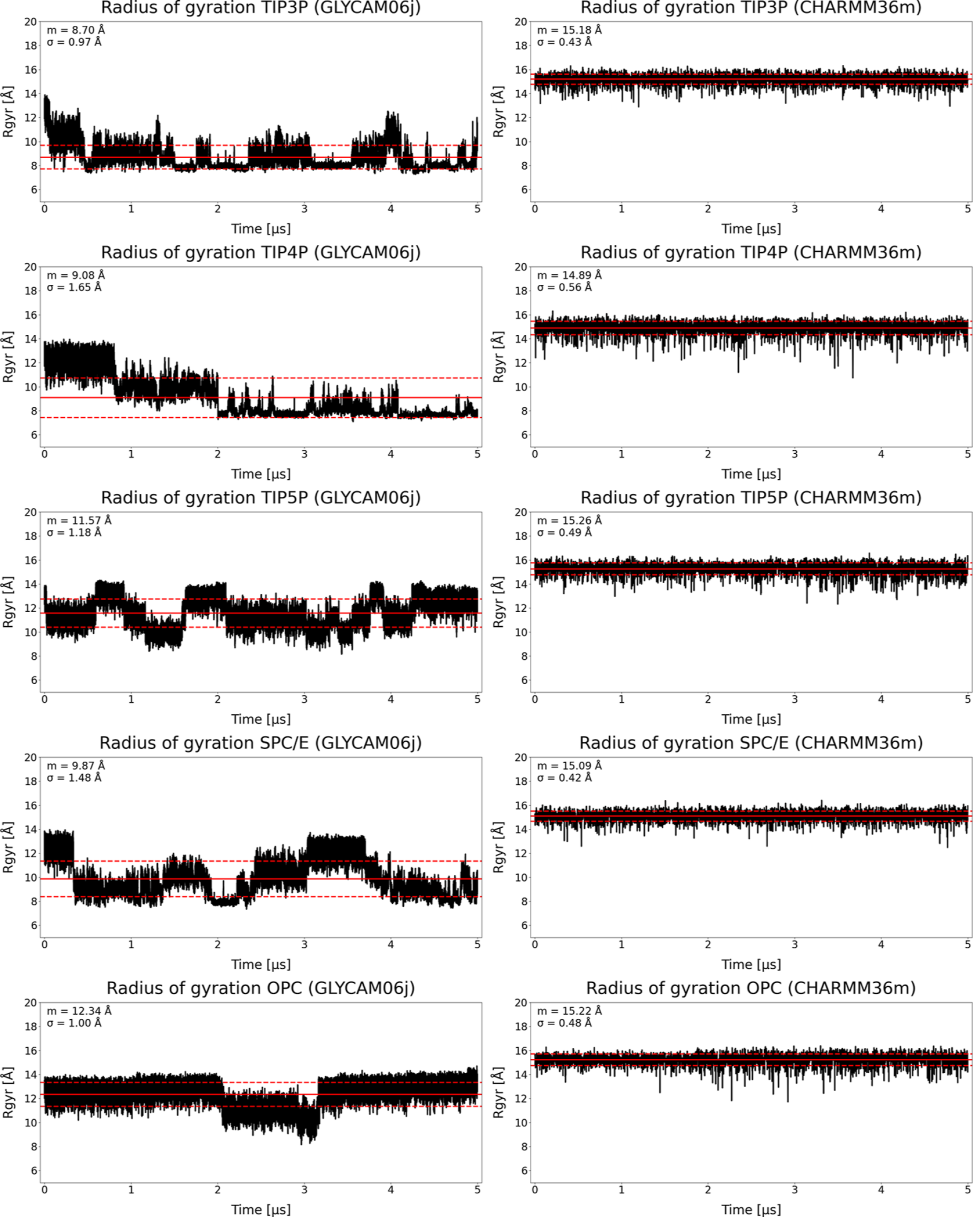
Radius of gyration results of HP dp10 in GLYCAM06j and HP dp12 in CHARMM36m, solvated in selected explicit solvent models.

Notably, both mean values and standard deviations of *R*_g_ in CHARMM36m align more closely with those reported for implicit solvent models using GLYCAM06j than with those obtained from equivalent explicit solvent simulations.^41^ For GLYCAM06j, the presence of multiple HP clusters was similarly reflected in RMSD matrices, with standard deviations in *R*_g_ typically two to three times higher than those observed for CHARMM36m. These findings suggest that HP exhibits greater conformational flexibility in GLYCAM06j than in CHARMM36m. The lower variability of *R*_g_ in CHARMM36m, despite explicit solvent treatment, implies that conformational dynamics are more restrained, resulting in a level of compactness comparable to that found under implicit solvent conditions. This contrast likely arises from differences in how solvent–solute interactions are represented in the two force fields, underscoring the importance of force field choice in simulations of GAG conformational behavior.

### Correlation Analysis of Structural Properties

To better understand the relationships among the investigated structural properties, correlation analyses were performed for all solvent model and force field combinations (Figure 7). Distinct trends emerged, particularly within the GLYCAM06j force field, where strong correlations were observed between RMSD and EED, especially for TIP3P, TIP4P, and SPC/E. In these cases, a decrease in EED was accompanied by an increase in RMSD, indicating that more compact conformations tend to deviate more from the initial structure. This relationship points to a higher degree of structural flexibility and conformational variability.

**Figure 7:**
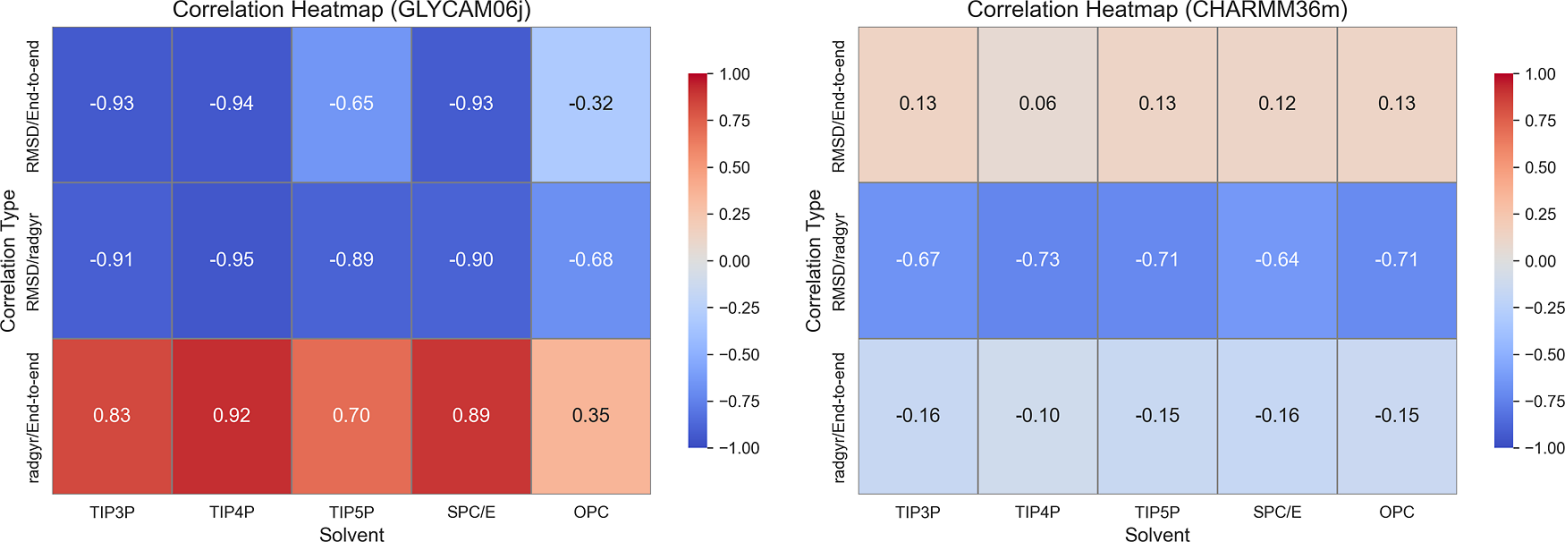
Correlation heatmaps of structural properties in GLYCAM06j and CHARMM36m force fields.

A similarly strong anticorrelation was observed between RMSD and *R*_g_ in GLYCAM06j. Structures that were more compact than the initial MD frame exhibited higher RMSD values, suggesting that these conformations, though closer in size to the reference, undergo significant rearrangements. Such behavior likely reflects bending or folding of the carbohydrate chain, leading to compact yet distorted configurations. Additionally, a strong positive correlation between *R*_g_ and EED was identified for all solvent models in GLYCAM06j, with the exception of OPC. This result suggests that more extended structures, with larger EED, also possess a greater spread of atomic mass around the center of mass.

In contrast, CHARMM36m yielded weaker and often opposite correlation patterns. A modest negative correlation between RMSD and EED was observed, implying that more extended conformations remain closer to the initial structure, indicative of reduced flexibility in this force field. Furthermore, the *R*_g_ showed a slight anticorrelation with EED, opposing the trend seen in GLYCAM06j. This suggests that in CHARMM36m, molecular extension is not strongly coupled to overall compactness. Such behavior may arise from local conformational effects, such as sulfate or carbohydrate group rotations, ring puckering, or variations in glycosidic linkage geometries. These subtle local modifications affect global dimensions only marginally, resulting in weak correlations.

Despite differences in overall trends, the correlation between RMSD and *R*_g_ was qualitatively similar for both force fields. While the magnitudes varied, both showed that compact structures tend to exhibit greater deviations from the reference frame, although the underlying causes may differ.

Taken together, these results indicate that the GLYCAM06j force field captures larger-scale conformational rearrangements, such as chain bending or extension, which manifest as strong correlations among global structural descriptors. In contrast, CHARMM36m tends to preserve the general molecular shape, with variability driven by more localized changes that exert less influence on global metrics such as *R*_g_ or EED.

### Ring Puckering Distribution

In this study, we also examined the ring conformational preferences of individual IdoA and GlcNS residues across selected solvent models (Figure 8). This aspect is particularly important, as previous work has demonstrated that ring puckering can influence the binding affinity of GAGs to their protein targets.^57^

**Figure 8:**
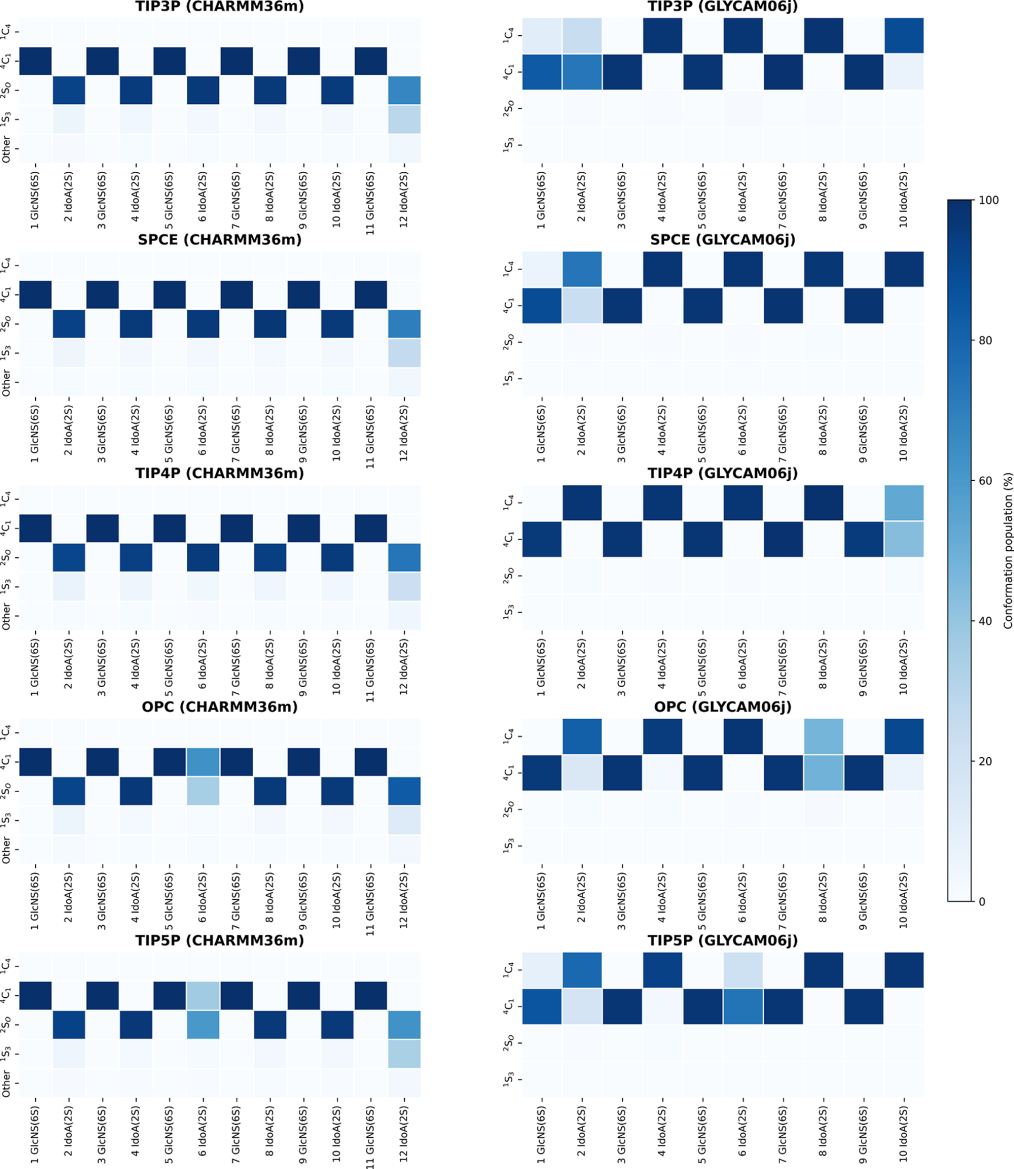
Distribution of monosaccharide ring conformations identified in MD simulations.

In general, for all solvent models studied in this system, the dominant conformation for all GlcNS­(6S) residues is ^4^C_1_. These results indicate that the choice of solvent model in CHARMM36m has no impact on the rigidity of this monosaccharide ring. The observed ring puckering distributions for GlcNS­(6S) are broadly consistent with those obtained using the GLYCAM06j force field, although minor differences are present. In particular, for selected residues, it was reported that GlcNS­(6S) may adapt the ^1^C_4_ conformation, which is not observed in the current study. This discrepancy may arise from a higher energetic barrier for the ^4^C_1_ → ^1^C_4_ transition in CHARMM36m relative to GLYCAM06j. Indeed, while we also identified the presence of other, noncanonical GlcNS­(6S) conformations in CHARMM36m, their contribution was insignificant (less than 1%).

For IdoA­(2S) residues, the most frequently observed ring conformation was ^2^S_O_, followed by ^1^S_3_, with the latter being most prevalent at the nonreducing end of the heparin chain. Interestingly, for the sixth IdoA­(2S) residue, we also observed the presence of the ^4^C_1_ conformation in simulations using the TIP5P and OPC solvent models. This suggests that both the solvent environment and the specific positioning within the heparin molecule may influence the conformational flexibility of this residue, enabling it to adapt a conformation not seen in the other positions. The ^4^C_1_ conformation was also present in GLYCAM06j to a greater extent. In this force field, regardless of the initial ring conformation of the IdoA­(2S) residue, simulations in the TIP3P solvent showed that both chair conformations were populated (with a preference for ^1^C_4_) and were generally more favored than skew conformations. This observation further supports the idea that the energy barriers for chair–skew transitions are higher in CHARMM36m than in GLYCAM06j and may require specific environmental conditions to be overcome.

On the other hand, the equilibrium between the ^2^S_O_ and ^1^S_3_ conformations, previously described for the GLYCAM06j force field, is also present to some extent in CHARMM36m. This suggests that both force fields exhibit similar capabilities in capturing the conformational energetics of IdoA­(2S) skew conformations, indicating a degree of consistency in their ability to model the structural dynamics of heparin.

### Glycosidic Linkage Analysis

Among the various properties used to describe the geometry of a carbohydrate chain, the glycosidic linkage stands out as a crucial descriptor. In this study, the results presented in Figure 9 were obtained by aggregating the populations of GlcNS­(6S)–IdoA­(2S) and IdoA­(2S)–GlcNS­(6S) glycosidic linkages across each of the water models investigated.

**Figure 9:**
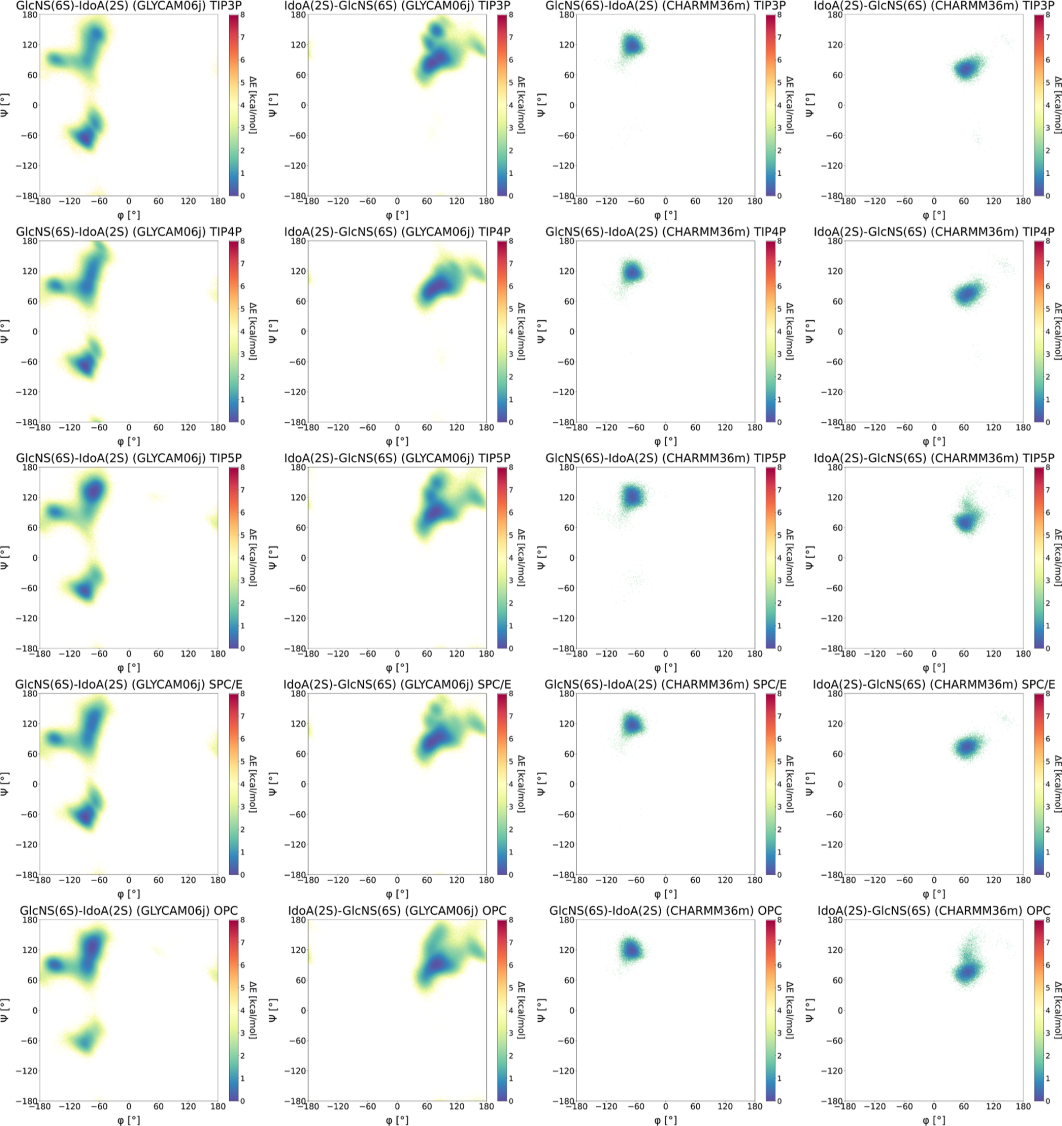
GlcNS­(6S)–IdoA­(2S) and IdoA­(2S)–GlcNS­(6S) glycosidic linkage heatmaps for ϕ and ψ dihedral angles in different solvent models in GLYCAM06j and CHARMM36m.

For all tested solvent models, the global minimum for the GlcNS­(6S)–IdoA­(2S) glycosidic linkage was located at ϕ ∼ −70° and ψ ∼ 120° (Figure 9). This observation is consistent with our previously reported 10 μs MD simulations of unbound HP dp6 in TIP3P,^15^ as well as with the results reported by Marcisz et al. for the respective solvent models using the GLYCAM06 force field.^41^ These findings also align with available experimental structures (PDB IDs: 1BFC, 1E0O, 1FQ9, 1G5N, 1GMN, 1QQP, 1RID, 1T8U, 1U4L, 1XMN, 1XT3, 2AXM, 2BRS, 2FUT, 2HYV, 2LVZ, 2NWG, 2WNU, 3DY0, 3INA, 3MPK, 3OGX, 3QMK, 4AK2, 4C4N, and 4DY0), in which the conformation of the GlcNS­(6S)–IdoA­(2S) linkage corresponds to this minimum. However, none of the MD simulations captured the previously reported secondary minimum at ϕ ∼ −150° and ψ ∼ 90°. Additionally, another local minimum, defined by ϕ ∼ −90° and ψ ∼ −50°, was only marginally sampled in the TIP5P model and, to an even lesser extent, in TIP3P, TIP4P, and SPC/E. This may indicate that the rotational barrier for transitioning from the global minimum to the local one is higher in CHARMM36m than in GLYCAM06j.

For the IdoA­(2S)–GlcNS­(6S) linkage, the effect of the solvent model on conformational flexibility was more pronounced (Figure 9). In general, we observed that increasing solvent model complexity led to an expanded conformational space. For all tested solvent models, the global minimum was located at ϕ ∼ 60° and ψ ∼ 60°. In the OPC and TIP5P models, an additional minimum was present at ϕ ∼ 60° and ψ ∼ 110°. A third minimum, at ϕ ∼ 140° and ψ ∼ 130°, was barely sampled in all solvent models, with slightly greater presence in SPC/E and TIP4P. These results are in agreement with those reported for GLYCAM06j. Notably, the minima identified in this study for the IdoA­(2S)–GlcNS­(6S) linkage are narrower than those described in the GLYCAM06j study, suggesting that the three distinct minima observed here may correspond to a single broader minimum in GLYCAM06j. This again points to a potentially higher rotational barrier for conformational transitions in CHARMM36m relative to GLYCAM06j.

## Conclusions

In this study, we evaluated the influence of explicit solvent models on the structural flexibility of HP. Analysis of RMSD for HP dp12 across five explicit solvent models reveals varying degrees of structural stability and flexibility. The TIP3P and SPC/E models demonstrate lower RMSD variability, indicating a consistently stable structural environment, whereas TIP4P exhibits increased variability, likely due to enhanced polarization effects. Comparison with GLYCAM06 results indicates that CHARMM36m effectively restrains conformational changes of HP. RMSD matrices corroborate these trends, with TIP3P and SPC/E showing stable structures, while TIP4P, OPC, and TIP5P display clusters of high RMSD values, suggesting increased structural variability in these solvents.

Analysis of EED and radius of gyration (*R*_g_) across solvent models for HP dp12 reveals consistent mean values and standard deviations, indicative of stable molecular extension and compactness. Changes in RMSD do not correlate with variations in EED, suggesting that structural dynamics are influenced by factors such as glycosidic linkage conformations and ring puckering. These findings align with previous studies on HP dp10, highlighting distinctions between CHARMM36m and GLYCAM06 in capturing molecular compactness and stability under explicit and implicit solvent conditions. Specifically, TIP3P and SPC/E maintain stable conformations, whereas TIP4P, OPC, and TIP5P exhibit variability potentially linked to solvent-induced structural changes observed in the *R*_g_ analysis.

TIP3P solvent model show consistent inter-residue distances across all investigated models, typically within ±1.2 Å. TIP4P favors more extended structures relative to TIP3P, while TIP5P induces a more compact arrangement, emphasizing structural stability at the potential cost of reduced flexibility. SPC/E closely resembles TIP3P in contact patterns, whereas OPC exhibits a mixed trend, with sequential residues showing increased distances and distant residues displaying closer contacts, suggesting a preference for compact configurations influenced by specific solvent interactions.

Glycosidic linkages of GlcNS­(6S)–IdoA­(2S) and IdoA­(2S)–GlcNS­(6S) in heparin were examined across various solvent models, revealing consistent global minima across all models—specifically, ϕ ∼ −70° and ψ ∼ 120° for GlcNS­(6S)–IdoA­(2S) and ϕ ∼ 60° and ψ ∼ 60° for IdoA­(2S)–GlcNS­(6S). These findings are consistent with prior molecular dynamics simulations and experimental data, demonstrating robustness across methodologies. However, certain local minima reported in other studies were absent here, suggesting differing energy barriers in CHARMM36m compared to GLYCAM06, highlighting the force field’s impact on glycosidic linkage conformations in heparin.

In conclusion, our comprehensive assessment of explicit solvent models on HP structural dynamics underscores their critical role in biomolecular simulations. TIP3P and SPC/E emerge as optimal choices for maintaining stable HP conformations, characterized by lower RMSD variability, consistent EED and *R*_g_ values, and lower computational costs compared to more advanced solvent models. Conversely, TIP4P, OPC, and TIP5P exhibit greater RMSD variability and diverse contact map patterns, suggesting enhanced flexibility potentially driven by solvent-induced effects. Comparison with GLYCAM06 highlights CHARMM36m′s efficacy in restraining HP conformational changes, despite observed differences in capturing specific glycosidic linkage conformations. This study provides valuable insights for selecting suitable solvent models to accurately study carbohydrate molecule dynamics and stability in biomolecular simulations.

## Data Availability

The data supporting this study are openly available in the Zenodo repository (10.5281/zenodo.17077612). The repository contains MD trajectories of HP dp12 in TIP3P, TIP4P, TIP5P, SPC/E, and OPC solvent models, together with the corresponding topology files and minimized structures.
